# Enhanced Pedestrian Navigation Based on Course Angle Error Estimation Using Cascaded Kalman Filters

**DOI:** 10.3390/s18041281

**Published:** 2018-04-21

**Authors:** Jin Woo Song, Chan Gook Park

**Affiliations:** 1 School of Intelligent Mechatronic Engineering, Sejong University, 209 Neungdong-ro, Gwangjin-gu, Seoul 05006, Korea; jwsong@sejong.ac.kr; 2 Department of Mechanical and Aerospace Engineering/ASRI, Seoul National University, 1 Gwanak-ro, Gwanak-gu, Seoul 08826, Korea

**Keywords:** pedestrian dead reckoning, zero velocity update, course angle error, two cascaded Kalman filters (TCKF), INS-EKF-ZUPT

## Abstract

An enhanced pedestrian dead reckoning (PDR) based navigation algorithm, which uses two cascaded Kalman filters (TCKF) for the estimation of course angle and navigation errors, is proposed. The proposed algorithm uses a foot-mounted inertial measurement unit (IMU), waist-mounted magnetic sensors, and a zero velocity update (ZUPT) based inertial navigation technique with TCKF. The first stage filter estimates the course angle error of a human, which is closely related to the heading error of the IMU. In order to obtain the course measurements, the filter uses magnetic sensors and a position-trace based course angle. For preventing magnetic disturbance from contaminating the estimation, the magnetic sensors are attached to the waistband. Because the course angle error is mainly due to the heading error of the IMU, and the characteristic error of the heading angle is highly dependent on that of the course angle, the estimated course angle error is used as a measurement for estimating the heading error in the second stage filter. At the second stage, an inertial navigation system-extended Kalman filter-ZUPT (INS-EKF-ZUPT) method is adopted. As the heading error is estimated directly by using course-angle error measurements, the estimation accuracy for the heading and yaw gyro bias can be enhanced, compared with the ZUPT-only case, which eventually enhances the position accuracy more efficiently. The performance enhancements are verified via experiments, and the way-point position error for the proposed method is compared with those for the ZUPT-only case and with other cases that use ZUPT and various types of magnetic heading measurements. The results show that the position errors are reduced by a maximum of 90% compared with the conventional ZUPT based PDR algorithms.

## 1. Introduction

The personal navigation system (PNS) technology, which provides absolute or relative navigation information such as position and orientation of a user, is an emerging technology widely used for location-based services (LBS), health care systems, future soldier systems, and many other application areas [1]. There are two main approaches for acquiring proper navigation information, a dependent PNS and a self-contained PNS [2]. The radio navigation systems such as the global navigation satellite system (GNSS) and wireless local area network (WLAN) based navigation system are the most widely used dependent PNSs which require external aiding signals or information [3,4]. As the dependent PNS depends on the infrastructure providing additional information, and this infrastructure is not available everywhere, a seamless solution cannot be easily obtained by using it.

The self-contained PNS is another approach for localization, and a personal dead reckoning (PDR) approach is one of the mostly used self-contained PNSs, which generally adopts an inertial sensor based navigation system [1,5,6,7]. Because it is not dependent on the information from infrastructures, a seamless solution available under almost every condition is achievable. However, the self-contained navigation sensors represented by inertial sensors such as accelerometers and gyroscopes induce significant position errors caused by their bias instability [8,9]. In order to minimize the position error, a step-length estimation approach has been developed. It uses inertial sensors for estimating stride length and magnetic sensors for measuring direction. The estimated step length is combined with the direction to determine the position [10,11,12]. This approach provides relatively accurate travel distance. However, the direction can be easily influenced by the external magnetic disturbances, which results in insufficient position accuracy.

The inertial navigation system (INS) approach, which uses an extended Kalman filter (EKF) and zero velocity updates (ZUPT) for estimating navigation and sensor error states, has also been applied for indoor pedestrian navigation applications [13,14,15,16,17], and many other applications recently [18,19,20,21]. This approach, which is generally called the INS-EKF-ZUPT approach, uses an inertial measurement unit (IMU) installed on a foot or shoe, and the characteristics of the walking gait cycle, which is composed of stance and swing phases. The stance phase can be classified into heel strike phase, foot flat phase, mid-stance phase, and push-off phase. During the foot flat and mid-stance phase, the velocity of the foot where an IMU is attached is assumed to be zero, and an EKF uses the zero velocity measurements for estimating the sensor bias errors, attitude errors, and velocity errors during the phases. The zero velocity measurements are very useful for velocity error estimation, and thus, the walking distance can be accurately estimated by using the measurements. However, the yaw gyro bias error and yaw angle error are not observable for the measurements because the zero velocity measurements do not contain information for direction. Thus, the position accuracy is highly influenced by the heading accuracy.

In order to improve the heading and position accuracy, a magnetic sensor installed in the IMU can be used. Because the magnetic heading measurements are used for estimating heading error and yaw gyro bias, the position accuracy can be improved in general compared with the simple step-length estimation approach [13,15]. Although the magnetic sensors can provide seamless heading information, they can be easily contaminated by external magnetic sources or magnetic substances. The hard or soft iron effects by the environmental disturbances distort the earth magnetic fields and prevent magnetic sensors from providing accurate heading information.

The integration of a dependent PNS and self-contained PNS has been developed in order to improve the position accuracy. A GNSS or indoor localization, based on WLAN, RFID, or other ranging methods, is integrated into the PDR [22,23,24,25,26,27]. Fingerprinting based navigation algorithms, which use WLAN, the magnetic environment, or a natural landmark [28,29,30] have also been proposed for improving position accuracy. Recently, map based navigation algorithms, which use a nonlinear filtering technique like the particle filter, have also been developed [31,32,33]. These approaches can provide a reliable solution for indoor positioning. However, they still rely on the infrastructure or pre-calculated map information significantly. Thus, they cannot be applied to general cases but to specific areas where infrastructures are installed or prerequisite information is acquired. Moreover, this research primarily focuses on special applications for people who perform special duties, such as future soldiers and firefighters. In these cases, a GPS is usually supposed to be unavailable or jammed, and infrastructure is not available. Moreover, prior information such as map data is not known in general, and thus, an inertial navigation becomes more significant. Therefore, pedestrian navigation based on inertial navigation should be the fundamental navigation system, even though it requires additional equipment and causes inconvenience to users. As there is no additional information available in these cases, more accurate heading information is a requisite.

To realize the fully self-contained navigation system providing more reliable position solution and heuristic approaches, which use the walking characteristics of pedestrians, have been developed [34,35,36], and the heuristic solution is integrated into the map data using a map matching technique [37]. Although they can provide a reasonable solution for most situations, they cannot handle the exceptional cases in which pedestrians do not walk in general walking patterns. Multiple sensor fusion approaches using the kinematic skeleton model of the human body have also been developed [15,38], which require many sensors for positioning, and therefore, impose excessive computational burden. In addition, research to improve the navigation performance of a low-cost integrated navigation system has been carried out in various fields, especially for unmanned vehicles [39,40,41,42]. These algorithms are optimized for each target application, and thus should be modified to be applicable to the pedestrian navigation systems.

In order to address these issues, a course-angle error estimation approach, which uses a simple kinematic relation and ZUPT based navigation system with cascaded Kalman filter architecture, is proposed in this paper. As the magnetic sensors on the waist position are likely to be less contaminated by the external magnetic disturbances, they are used for estimating the sensor heading error of an IMU mounted on a foot. The measurements from the magnetic sensors, however, cannot be used directly for the sensor-heading error estimation because there is a difference between the course angle and sensor heading, which is defined as the toe-in or toe-out angle, and is not negligible [43,44]. In order to determine the relation between the course angle and sensor heading, a simple kinematic toe angle model is used in this study. Under the kinematic constraints, the course angle error can be assumed to have the same average value as the sensor heading error, although the course angle is different from the sensor heading by the toe angle. For estimating the course angle error, the magnetic heading and measured course angle are used. The measured course angle is inferred by using the current and one-stance-before positions from the INS-EKF-ZUPT solution of a foot-mounted IMU. As the course angel error and sensor heading error may have different stochastic characteristics, except their means, the estimated course angle error cannot be used directly for the sensor heading error. Thus, a cascaded Kalman filter structure is used, and the course angle error estimated at the first state is used as measurement for estimating the sensor heading error at the second stage. The proposed approach can provide a reliable and case insensitive solution because it uses less contaminated magnetic data, and because it does not use the toe angle itself but the kinematic constraint and assumption of the analogy between the course angle error and sensor heading error.

In the next section, the conventional ZUPT-based PDR algorithm is introduced first. The stance-phase detection algorithm and the system model are described, and it is shown that the magnetic sensors on the waist can be less contaminated by magnetic disturbances compared with those in a foot-mounted IMU. The geometry and kinematic relation of the walking gait and trances are illustrated next, and the overall algorithm structure is explained in more detail. The filter structures and filter models are proposed in the next section, and time propagation and measurement propagation of covariance are explained. The proposed algorithm is validated by the experimental results, and error analyses are performed by using a new concept of way-point position error (WPE).

## 2. Zero Velocity Update (ZUPT) Based Pedestrian Dead Reckoning System

**Figure 1:**
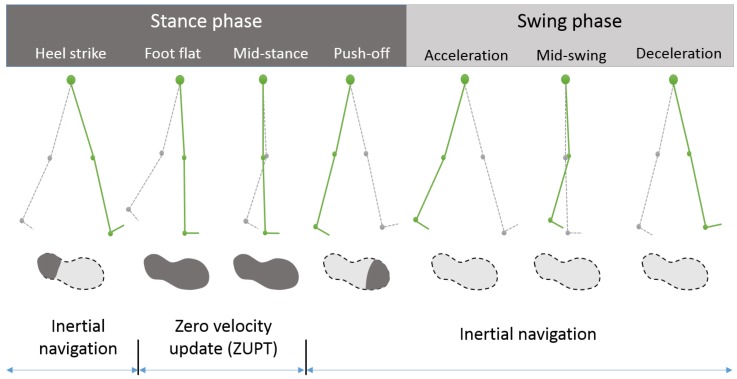
Walking gait cycle and measurement update cycle.

In indoor pedestrian localization systems, PDR is a widely used technique, which applies an integration-based navigation algorithm and the ZUPT method [45]. In a PDR system, a foot-mounted IMU is used for detecting the position and orientation, and the errors are compensated by using the ZUPT method. The ZUPT technique is based on the characteristics of the walking gait of humans. The walking cycle of a human can be divided into two parts, the stance and swing phases, as shown in Figure 1 [16,46]. During the stance phase, the sole of a foot is in contact with the ground partially or entirely. In the foot flat and mid-stance phases, it can also be assumed that the foot adheres to the ground thoroughly, which means that the foot does not move during these phases. Thus, we can assume that the velocity of the foot is zero during these phases, which gives zero velocity measurements for a filter or system. Using these measurements, a system can reset the navigation information or update error states.

For obtaining the navigation information, such as orientation, velocity, and position, the navigation system adopts the INS-EKF-ZUPT algorithm, which includes the inertial navigation algorithm basically for localization and the EKF for estimating error states using the zero velocity measurements. In order to design the INS-EKF-ZUPT system, the stance phases should be detected first. In order to detect the stance phases, the raw data from accelerometers and gyroscopes are used. As the foot stands sound during the stance phase, the foot-mounted IMU experiences sound and still motion, which causes the sensor outputs to have small variances. Thus, the stance phases can be detected by comparing the variation of the accelerations and that of the angular rates with some thresholds [6].

The variation of the accelerations at time step *k* for time period $k_{0}$ can be defined in three ways; the variances of energy, $E_{a}$, product, $P_{a}$, and sum, $S_{a}$ [15].

(1) $$E_{a}=\mathrm{Var}\left(\sqrt{{\left(a_{x}\left(k-k_{0}\right)\right)}^{2}+{\left(a_{z}\left(k-k_{0}\right)\right)}^{2}},\cdots ,\sqrt{{\left(a_{x}\left(k\right)\right)}^{2}+{\left(a_{z}\left(k\right)\right)}^{2}}\right)$$

(2) $$P_{a}=\mathrm{Var}\left(a_{x}\left(k-k_{0}\right)a_{z}\left(k-k_{0}\right),\cdots ,a_{x}\left(k-1\right)a_{z}\left(k-1\right),a_{x}\left(k\right)a_{z}\left(k\right)\right)$$

(3) $$S_{a}=\mathrm{Var}\left(a_{x}\left(k-k_{0}\right)+a_{z}\left(k-k_{0}\right),\cdots ,a_{x}\left(k-1\right)+a_{z}\left(k-1\right),a_{x}\left(k\right)+a_{z}\left(k\right)\right)$$

Here, $a_{x}\left(k\right)$ and $a_{z}\left(k\right)$ denote the acceleration along the *x* axis and *z* axis at time step *k*, respectively. As the acceleration along the *y* axis is not significant for pedestrian navigation, it is ignored. The variation of the angular rates can be defined in two ways: the variance of energy, $E_{r}$, and energy at time step *k*, $A_{r}$ as follows [15]:

(4) $$E_{r}=\mathrm{Var}\left(\sqrt{{\left(\omega_{x}\left(k-k_{0}\right)\right)}^{2}+{\left(\omega_{y}\left(k-k_{0}\right)\right)}^{2}},\cdots ,\sqrt{{\left(\omega_{x}\left(k\right)\right)}^{2}+{\left(\omega_{y}\left(k\right)\right)}^{2}}\right)$$

(5) $$A_{r}=\sqrt{{\left(\omega_{x}\left(k\right)\right)}^{2}+{\left(\omega_{y}\left(k\right)\right)}^{2}},$$

where $\omega_{x}\left(k\right)$ and $\omega_{y}\left(k\right)$ are the angular rates along the *x* and *y* axes, respectively. In this study, the time period for calculating the variances is set to 15 samples which is equivalent to 0.15 s for 100 Hz sampling rate. The sampling rate of 100 Hz is chosen to detect the large and momentary acceleration experienced at the heel strike phase [16]. When the values of all these indicators are less than some thresholds, the stance phase is declared and a zero velocity state is assumed. That is, a stance phase is detected when the indicator *J* is less than one, as follows:

(6) $$J=\max\left(w_{1}E_{a},w_{2}P_{a},w_{3}S_{a},w_{4}E_{r},w_{5}A_{r}\right)\leq 1,$$

where $w_{i}$ (i=1,⋯,5) is a weighting factor for each index. In this paper, $w_{1}=50$, $w_{2}=w_{3}=100$, $w_{4}=3$, and $w_{5}=5$, which are determined empirically.

In order to calculate the position, velocity, and orientation of the foot-mounted IMU, an inertial navigation algorithm based on quaternion is used. As the navigation solution from an INS is prone to diverge without additional measurements, the ZUPT at the stance phase is applied for suppressing the diverging characteristics. For estimating the error states and compensating them, an EKF with 12 error states, as defined in (7), is used.

(7) $$x={\left[\begin{array}{cccc} \delta \phi^{T} & \delta v^{T} & b_{g}^{T} & b_{a}^{T} \end{array}\right]}^{T}$$

Here, $\delta \phi ={\left[\begin{array}{ccc} \delta \phi_{N} & \delta \phi_{E} & \delta \phi_{D} \end{array}\right]}^{T}$ is an orientation error state vector in local navigation, north-east-down (NED) frame, $\delta v={\left[\begin{array}{ccc} \delta v_{N} & \delta v_{E} & \delta v_{D} \end{array}\right]}^{T}$ is a velocity error state vector, $b_{g}={\left[\begin{array}{ccc} b_{g,N} & b_{g,E} & b_{g,D} \end{array}\right]}^{T}$ is a gyroscope bias vector, and $b_{a}={\left[\begin{array}{ccc} b_{a,N} & b_{a,E} & b_{a,D} \end{array}\right]}^{T}$ is an accelerometer bias vector. Because the zero velocity measurements are used for the EKF, the position error is not fully observable. Hence the position error state is omitted for the EKF.

The error propagation model and measurement model for this case can be obtained using the linear perturbation method. Noting that the PNS can be considered as a local level navigation system, and that the sensors used in the system are not precise enough to detect the earth rate, the earth rate applied to the system, the Coriolis term, and the gravitational error can be neglected. In this case, the error propagation model and measurement model can be simplified as follows [8,9,47]:

(8) $$\dot{x}=\mathrm{Fx}+\mathrm{Gw}=\left[\begin{array}{cccc} 0_{3\times 3} & 0_{3\times 3} & -C_{b}^{n} & 0_{3\times 3}\\ S & 0_{3\times 3} & 0_{3\times 3} & C_{b}^{n}\\ 0_{3\times 3} & 0_{3\times 3} & -\beta_{g}I_{3\times 3} & 0_{3\times 3}\\ 0_{3\times 3} & 0_{3\times 3} & 0_{3\times 3} & -\beta_{a}I_{3\times 3} \end{array}\right]x+\left[\begin{array}{cccc} -C_{b}^{n} & 0_{3\times 3} & 0_{3\times 3} & 0_{3\times 3}\\ 0_{3\times 3} & C_{b}^{n} & 0_{3\times 3} & 0_{3\times 3}\\ 0_{3\times 3} & 0_{3\times 3} & I_{3\times 3} & 0_{3\times 3}\\ 0_{3\times 3} & 0_{3\times 3} & 0_{3\times 3} & I_{3\times 3} \end{array}\right]w$$

(9) $$z=\mathrm{Hx}+\nu =\left[\begin{array}{cccc} 0_{3\times 3} & I_{3\times 3} & 0_{3\times 3} & 0_{3\times 3} \end{array}\right]x+\nu ,$$

where $w={\left[\begin{array}{cccc} n_{g}^{T} & n_{a}^{T} & \omega_{g}^{T} & \omega_{a}^{T} \end{array}\right]}^{T}$ is an input noise vector. Here $n_{g}$ and $n_{a}$ are white Gaussian noise vectors for gyroscopes and accelerometers with power spectral densities $\sigma_{g}^{2}I$ and $\sigma_{a}^{2}I$, respectively, and $\omega_{g}$ and $\omega_{a}$ are white Gaussian noise processes for the sensor bias models with power spectral densities $\sigma_{wg}^{2}I$ and $\sigma_{wa}^{2}I$, respectively. ν is a measurement noise vector whose covariance matrix is R, $C_{b}^{n}$ is a rotation matrix, and S is a skew symmetric matrix representing the vector cross product by the gravity-compensated accelerations in the NED frame, $a_{N}$, $a_{E}$, and $a_{D}$.

(10) $$S=\left[\begin{array}{ccc} 0 & -a_{D} & a_{E}\\ a_{D} & 0 & -a_{N}\\ -a_{E} & a_{N} & 0 \end{array}\right].$$

**Figure 2:**
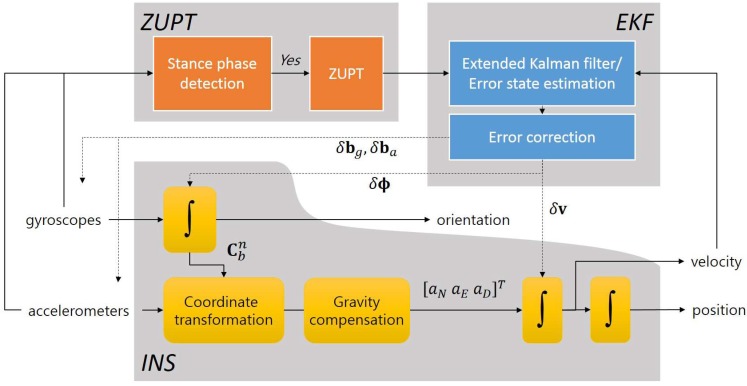
INS-EKF-ZUPT algorithm flow chart.

In this work, the gyroscope and accelerometer biases are assumed to be first order Gauss–Markov processes with the large time constants $\beta_{g}^{-1}$ and $\beta_{a}^{-1}$, respectively. The error states defined in (7) are estimated by a standard EKF with zero velocity measurement during the stance phases. The estimated error states are used for correcting the sensor and navigation errors. The overall structure and flow of an INS-EKF-ZUPT algorithm is illustrated in Figure 2.

Because the zero velocity measurements are very strong and reliable measurements for estimating the velocity errors and biases of accelerometers, the travel distance is likely to be sufficiently accurate, which means that only the heading or yaw angle accuracy is a dominant factor for position accuracy in the INS-EKF-ZUPT navigation system. Although the yaw angle error is included in the error states, the yaw angle error is not observable in a standard INS-EKF-ZUPT algorithm because the zero velocity measurements cannot define the heading or yaw angle. Thus, the position is prone to be distorted in spite of the accurate travel distance, which eventually degrades the position accuracy.

In order to acquire the observability for the yaw angle or heading error state, new measurements that can provide information about the heading angle need to be used. If the position measurements such as position from GPS are available, the yaw-angle error state becomes observable. For the indoor navigation application, unfortunately, the position measurements are supposed to be unavailable in many cases. Thus, other measurements should be used, and the magnetic sensor, which measures the earth magnetic field, is one of the best solutions.

The magnetic environment on or just above the ground, however, is liable to be contaminated. High voltage lines or reinforcing rods installed under the ground or floor induce hard iron or soft iron effects and distort the geomagnetic field, which results in the magnetic heading error. Nonetheless, the geomagnetic field near the waist is supposed not to be contaminated harshly by magnetic substances or other nearby metals under the ground or floor. Although magnetic substances, such as those in a cellular phone or watch, can also affect the magnetic field, their effects are less significant or even negligible. Sometimes their effects can be counted as a constant and time invariant magnetic source, which can be pre-calibrated.

**Figure 3:**
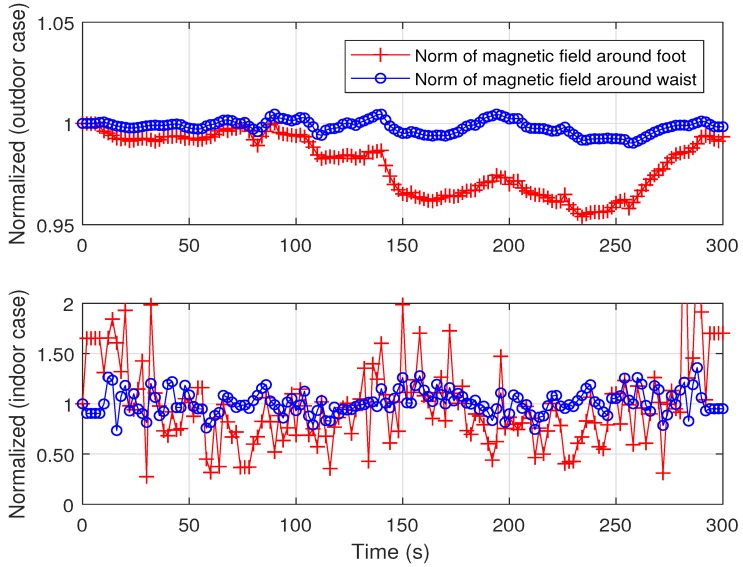
Normalized magnetic norm around foot (red line with ‘+’ markers) and waist (blue line with ‘o’ markers).

Figure 3, which presents data for demonstrating the magnetic disturbances around the waist and foot, shows these phenomena very well. The two lines represent the norms of the geomagnetic field at the waist and foot. In the experiments, magnetic sensors were installed at the waist and foot of an experimenter. The upper figure shows the results for an outdoor case. In this case, the experimenter strolled along the track for 300 s. As the geomagnetic field is normalized at the initial stage, the norms are expected to be one if there is no external magnetic disturbance. The figure shows that the norm of the magnetic field at the waist remains around one, as expected. The magnetic norm at the foot, however, fluctuates by the external magnetic disturbances, and the fluctuation causes magnetic heading error that cannot be condoned. The standard deviation of the magnetic norm at the waist is 0.0033, but that on the ground is 0.0142. The lower figure shows the results for an indoor case, which shows the same results as the outdoor case. While the standard deviation of the magnetic norm on the ground is 0.4701, at the waist it is 0.1183. In the indoor case, the magnetic error even at waist height is large, but is not biased and still tolerable if the measurement noise covariance is well adjusted. The results imply that the geomagnetic field measured at the waist is more reliable against disturbance.

This claim has been proven through numerous experiments, and the results are shown in Table 1. The experiment was carried out in 50 indoor sites and nine sets of data were obtained for each site. In conclusion, in total 450 sets of data were utilized for analyzing the magnetic heading error around the waist and on the floor. The experimental sites were classified into four categories: office buildings, home buildings, shopping malls or large halls, and underground spaces. Office building data include data from corridors, offices, parking buildings, meeting rooms, and stairs. Home building data include data from rooms, living rooms, kitchens, toilets, and terraces. Mall data are obtained from shopping malls, large lecture rooms, and the lobby. Underground data are obtained from subway stations, underground parking lots, basements, and so on.

The results in Table 1 show indoor magnetic heading errors around the waist and ground. The magnetic heading around the foot has a significant error of more than 17${}^{\circ }$, which is not suitable for the Kalman filter as a measurement. On the other hand, the error around the waist is not as large as the error around the foot. Experimental results show that the root-mean-square (RMS) error around the foot is about 2.5 times larger than that around the waist. Especially, the angle error experimented on the stairs was the smallest. The RMS error around the waist was 4.308${}^{\circ }$ and that around the foot was 8.426${}^{\circ }$. From the experimental results, we can conclude that the magnetic condition and heading accuracy are much better in the stair sections. The reason is that there are no obstacles in the stair sections which induce the magnetic disturbances. Therefore, it is expected that similar results will be obtained irrespective of whether the experimental path is two-dimensional or three-dimensional.

Although the magnetic heading measured around the waist can be used for the Kalman filter by adjusting the covariance of the measurement noise, it is still difficult to apply it directly because it is not negligibly small.

Further, it cannot be directly used as a new measurement for the EKF because there is an angle difference between the foot-mounted IMU and the waist-mounted magnetic sensors. In most cases, the difference has not been considered significant, and sometimes, it has been ignored. Actually, the difference can be ignored if the relative position is of interest. As the error is assumed to be a constant bias, it may cause only a rotation of the overall trajectory, and thus, it does not affect the relative position accuracy. However, the error causes an absolute position error owing to the heading error. In order to reduce the absolute position error, a new approach for estimating the angle difference should be applied, which is the main contribution of this study and is explained in the following sections.

## 3. Cascaded Kalman Filter Architecture for Course Angle Error and Heading Estimation

### 3.1. Geometry and Algorithm Architecture

For improving the observability of the heading error state, a new heading measurement from magnetic sensors is necessary and the waist-mounted magnetic sensors can provide less contaminated information, as mentioned in the previous section. However, the angle difference between the waist-mounted magnetic sensors and the foot-mounted IMU should be compensated for accurate positioning. If the magnetic sensors are mounted above the non-ferromagnetic buckle or backside, and if there is no toe-in or toe-out angle, the angle difference can be ignored and the magnetic heading can be counted as the heading of the foot-mounted IMU. In fact, the magnetic sensors and body can be sufficiently aligned to ignore the misalignment error. The toe-in or toe-out angle, however, cannot be ignored because it occurs naturally by the walking behavior of a human. People may have a toe angle of up to 15${}^{\circ }$, and the toe angles depend on walking speed and walking conditions, which means that the toe angle should be compensated but it is not easily compensable [43,44]. Because of the toe angle, the magnetic heading cannot be directly used as the heading measurements for the foot-mounted IMU. If it is used directly, it causes an angle shift that is related to the toe angle. Thus, toe angle estimation is required for an accurate solution when the misalignment error of the magnetic sensor is ignored.

Although the toe angle can be estimated using the heading measurements from the waist-mounted and foot-mounted magnetic sensors, the toe angle estimation is not suitable for enhancement of the heading accuracy because of the magnetic disturbance contaminating the foot-mounted magnetic sensors. In this case, the magnetic disturbance degrades significantly the quality of the measurements, which results in the heading estimation error. Thus, a novel approach for estimating the heading error is inevitable for absolute position accuracy.

**Figure 4:**
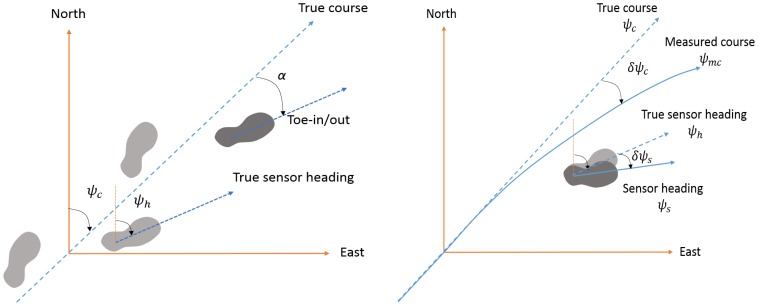
Geometry and kinematic relation for walking gait and traces.

Before introducing the proposed approach, the geometry and kinematic relation for the human walking gait is defined as shown in Figure 4 [46]. In this case, it is assumed that waist-mounted magnetic sensors are aligned to the walking direction, or course. In this figure, the course angle $\psi_{c}$ is not identical to the sensor heading angle $\psi_{h}$ of a foot-mounted IMU when this IMU is aligned to the direction of the foot nose. The angle difference is caused by the toe-in or toe-out angle α. The sensor heading $\psi_{s}$ includes errors due to the gyroscope bias and initial heading error, which is denoted by $\delta \psi_{s}$, thus it is different from the true heading angle of the foot-mounted IMU, $\psi_{h}$, as follows:

(11) $$\delta \psi_{s}=\psi_{h}-\psi_{s}.$$

Because the gyroscopes have bias and scale factor errors, the measured course can be different from true course. If gyroscope errors are negligible, the measured course may be the same as the true course, which is not the general case. Letting $\psi_{mc}$ be the measured course angle, the course angle error, $\delta \psi_{c}$, can be defined as

(12) $$\delta \psi_{c}=\psi_{c}-\psi_{mc}.$$

If the foot-mounted IMU is rigidly installed and the skeleton model of a human body is not time varying for a short period, we can assume that the course angle error $\delta \psi_{c}$ is dominated by the sensor heading error $\delta \psi_{s}$, and that their long term characteristics are similar enough for us to ignore the differences. Thus, it can be assumed that the estimation of the sensor heading error, $\delta \hat{\psi_{s}}$, has the same mean as the estimation of the course angle error, $\delta \hat{\psi_{c}}$, although they may have different variances.

(13) $$E\left(\delta \hat{\psi_{s}}\right)=E\left(\delta \hat{\psi_{c}}\right).$$

The relation between these two errors is the key idea of the proposed algorithm. In order to enhance the heading and position accuracy against the magnetic disturbance, the sensor heading error should be estimated properly, and the relation between the course angle error and the sensor heading error implies that the estimated course angle error can be used for estimating the sensor heading error. Noting that two error states have different variances, the estimated course angle error cannot replace the estimation of the sensor heading error directly. Instead, it can be used as a measurement for estimating the sensor heading error. In this case, the variance of the estimated course angle error will be the variance of a measurement. As the proposed algorithm does not use the information of the toe angle itself, it can provide case insensitive solutions. That is, the proposed algorithm provides robust solutions against the installation error, walking gait variations, and toe angle differences among people.

### 3.2. Two Cascaded Kalman Filters (TCKF) for Enhanced Heading Error Estimation

**Figure 5:**
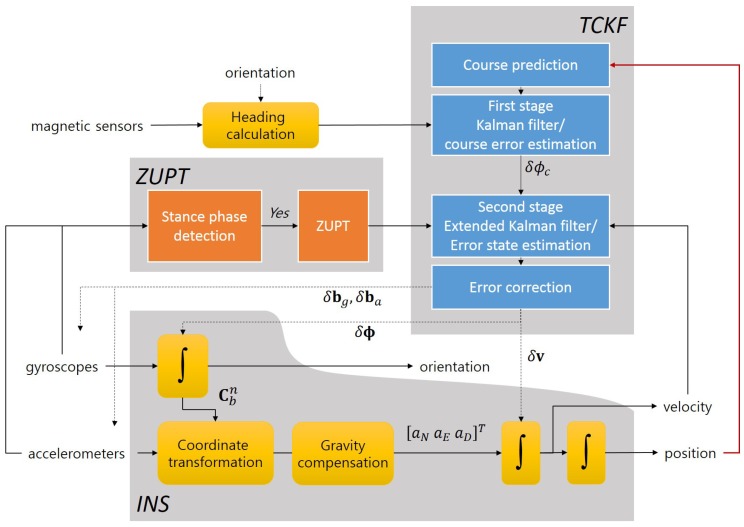
Proposed PDR structure with TCKF for course angle error estimation.

To realize the proposed concept, the two cascaded Kalman filters (TCKF) are used in this work. The cascaded Kalman filter structure has been used for reducing computational burden or for estimating state variables separately to obtain accurate estimates [48,49,50]. In this study, the cascaded structure is used for sequential estimations of error states that have similar characteristics and stochastic properties, and it enables the enhanced heading error estimation. In the first stage filter, the course angle error is estimated by using the measurements from magnetic sensors installed on the waist and the measured course angle, and the measured course angle is generated out of the trace of the position from the INS. The second stage filter uses the ZUPT information and estimated course angle error as measurements, and it estimates the navigation and sensor errors such as the attitude errors, velocity errors, gyroscope biases, and accelerometer biases. The estimated error states are used for correction. Because the proposed algorithm uses the cascaded Kalman filter structure, a new state variable is not augmented to the original Kalman filter, which does not significantly increase the computational cost. The basic architecture is shown in Figure 5.

As the course angle error is estimated alone at the first state, the error state of the first stage Kalman filter, $x_{1}$, becomes

(14) $$x_{1}=x_{1}=\delta \psi_{c}.$$

The course angle error is modeled as a first order Gauss-Markov process with the time constant $\beta_{c}^{-1}$ as

(15) $${\dot{x}}_{1}=-\beta_{c}x_{1}+n_{1},$$

where $n_{1}$ is the white Gaussian noise with variance of $\sigma_{1}^{2}$. The measurement equation of the first stage filter is

(16) $$z_{1}=H_{1}x_{1}+\nu_{1}=x_{1}+\nu_{1},$$

where $\nu_{1}$ is the measurement noise with variance of $\sigma_{c}^{2}$. The measurement for the course angle error estimation is generated from the difference between the heading from the magnetic sensors on the waist and the measured course angle. In order to obtain the measured course angle, the one-stance-before position of the foot-mounted IMU is used together with the current-stance position. Letting $s_{i}={\left[\begin{array}{cc} s_{N,i} & s_{E,i} \end{array}\right]}^{T}$ be the two dimensional position at the *i*-th stance phase, the measured course angle can be calculated as

(17) $$\psi_{mc}=\mathrm{atan}2\left(s_{i}-s_{i-1}\right)=\mathrm{atan}2\left(s_{N,i}-s_{N,i-1},s_{E,i}-s_{E,i-1}\right).$$

Hence the measurement can be obtained as

(18) $$z_{1,m}=\psi_{w}-\psi_{mc}=\psi_{w}-\mathrm{atan}2\left(s_{N,i}-s_{N,i-1},s_{E,i}-s_{E,i-1}\right),$$

where $\psi_{w}$ is the heading from the waist-mounted magnetic sensors.

The variance of the process noise, $\sigma_{1}^{2}$, is set to be slightly larger than $\sigma_{g}^{2}$ because it is mainly caused by the gyroscope noise. Because the measurement is related to the measured course angle out of the traces and magnetic heading of the waist-mounted sensors, the noise characteristics of the measurement are dependent on those of the positions and magnetic heading. Hence, the variance of the measurement noise can be simply assumed as

(19) $$\sigma_{c}^{2}=\gamma \sigma_{mag}^{2},$$

where γ is a positive constant which is a design parameter, and $\sigma_{mag}^{2}$ is the variance of the measurement noise in the magnetic heading.

Using the estimate from the first stage filter and ZUPT approach, the second stage filter estimates the navigation error states. In the second stage filter, the state vector and system model are the same as those of the conventional INS-EKF-ZUPT algorithm defined in (7) and (8).

(20) $$x_{2}={\left[\begin{array}{cccc} \delta \phi^{T} & \delta v^{T} & b_{g}^{T} & b_{a}^{T} \end{array}\right]}^{T}$$

(21) $${\dot{x}}_{2}={\mathrm{Fx}}_{2}+\mathrm{Gw}=\left[\begin{array}{cccc} 0_{3\times 3} & 0_{3\times 3} & -C_{b}^{n} & 0_{3\times 3}\\ S & 0_{3\times 3} & 0_{3\times 3} & C_{b}^{n}\\ 0_{3\times 3} & 0_{3\times 3} & -\beta_{g}I_{3\times 3} & 0_{3\times 3}\\ 0_{3\times 3} & 0_{3\times 3} & 0_{3\times 3} & -\beta_{a}I_{3\times 3} \end{array}\right]x_{2}+\left[\begin{array}{cccc} -C_{b}^{n} & 0_{3\times 3} & 0_{3\times 3} & 0_{3\times 3}\\ 0_{3\times 3} & C_{b}^{n} & 0_{3\times 3} & 0_{3\times 3}\\ 0_{3\times 3} & 0_{3\times 3} & I_{3\times 3} & 0_{3\times 3}\\ 0_{3\times 3} & 0_{3\times 3} & 0_{3\times 3} & I_{3\times 3} \end{array}\right]w$$

The measurement model for the second stage filter is different from that of a conventional INS-EKF-ZUPT based algorithm and becomes (22). Hence, a measurement becomes (23) because the estimated course angle error, ${\hat{x}}_{1}=\delta \psi_{c}$, is also used as a measurement together with zero velocity measurements.

(22) $$z_{2}=H_{2}x_{2}+\nu_{2}=\left[\begin{array}{cccc} \left[\begin{array}{ccc} \tan\phi_{N}\cos\phi_{D} & \tan\phi_{N}\sin\phi_{D} & -1 \end{array}\right] & 0_{1\times 3} & 0_{1\times 3} & 0_{1\times 3}\\ 0_{3\times 3} & I_{3\times 3} & 0_{3\times 3} & 0_{3\times 3} \end{array}\right]x_{2}+\nu_{2},$$

(23) $$z_{2,m}={\left[\begin{array}{cc} {\hat{x}}_{1} & \left[\begin{array}{ccc} 0 & 0 & 0 \end{array}\right] \end{array}\right]}^{T}$$

Here, the covariance matrix $R_{2}$ of the measurement noise $\nu_{2}$ can be chosen as

(24) $$R_{2}=\left[\begin{array}{cc} \sigma_{h}^{2} & 0_{1\times 3}\\ 0 & R \end{array}\right],$$

where $R=\sigma_{z}^{2}I_{3\times 3}$ is the covariance matrix for the zero velocity measurements, and $\sigma_{h}^{2}$ is the variance of the measurement noise. As the measurement ${\hat{x}}_{1}=\delta {\hat{\psi }}_{c}$ is the estimated value of $x_{1}=\delta \psi_{c}$, the variance of the measurement noise becomes the variance of the error state in the first stage filter as

(25) $$\sigma_{h}^{2}=P_{1}=\left[p_{1}\right],$$

where $P_{1}=\left[p_{1}\right]$ is the variance of the course angle error state in the first stage filter. At the error correction stage, all the navigation information, including the course angle error, is updated and corrected.

## 4. Experimental Results

**Figure 6:**
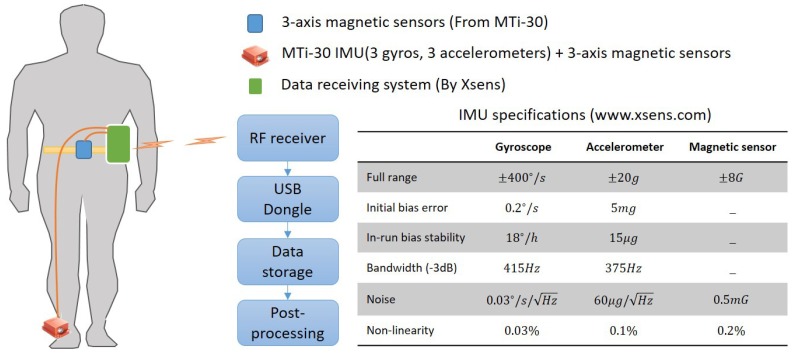
Measurement systems for indoor/outdoor experiments.

In this study, the effectiveness of the proposed algorithm is verified through experiments. In the experiments, three micro-electromechanical system (MEMS) based IMUs, the MTi-30 by Xsens Inc. consisting of three gyroscopes, three accelerometers, and three magnetic sensors, are used as the foot-mounted IMUs and waist-mounted magnetic sensors. The block diagram of the overall measurement system and the specifications of the IMUs are shown in Figure 6. For the experiments, we used accelerometers with the full measuring range of ±20 g because excessive acceleration can be detected during heel strike phase. For the same reason, IMUs with wide bandwidth were used.

The IMUs around the foot and waist transmit data to a data receiving system developed by Xsens. All data is time-synchronized, and gathered at 100 Hz. The data receiving system includes a battery pack that supplies power to the IMUs. The gathered data is transmitted to the RF receiver and USB dongle connected to a computer wirelessly. Before each experiment, an initialization time of about 5 s is required for initial alignment because each gyroscope has the initial bias error as shown in the specification table. The proposed algorithm was applied through post-processing.

All the experiments are performed outdoors and indoors for acquiring an accurate reference position. In order to investigate the effect of the toe angles and to compare the positions from the left and right feet, an IMU is mounted on the left and right feet, respectively. Because the same reference position can be used in spite of the different error characteristics and different toe angles of the left and right feet, the proposed experimental method is useful to validate the proposed algorithm and confirm the performance improvement.

In this work, the proposed algorithm, the TCKF case, is compared with the other three cases. In the ZUPT-only case, the zero velocity measurements at the stance phases are used only for estimating and correcting the errors. Thus, a foot-mounted IMU is only used in this case. In the ZUPT + sensor heading case, the sensor heading measurements from the magnetic sensors in the foot-mounted IMU are used for measurements together with the zero velocity measurements. Therefore, this case uses only one IMU mounted on the foot. In the ZUPT + course case, the course angle measured by the magnetic sensors in the waist-mounted IMU is used for estimating and correcting the heading error in addition to the ZUPT method. This case is theoretically identical to the case when the toe angle is ignored.

In order to compare the performances between the four cases, the return position error (RPE) and way-point position error (WPE) are calculated. Although the RPE is an easily used indicator in cases where an accurate position reference is not available, it cannot be a proper indicator in general because the position errors along the route are prone to be ignored. Thus, the WPE concept, which uses position references at several way-points for evaluating the position error, is used in this work. Because it uses several way-points on the route as reference points, it can report the position error along the route adequately if enough way-points are properly selected. Moreover, it can also be used indoors where not all the reference positions are obtainable generally. In the experiments, the inflection points where the walking direction changes are selected as way-points.

### 4.1. Outdoor Cases

**Figure 7:**
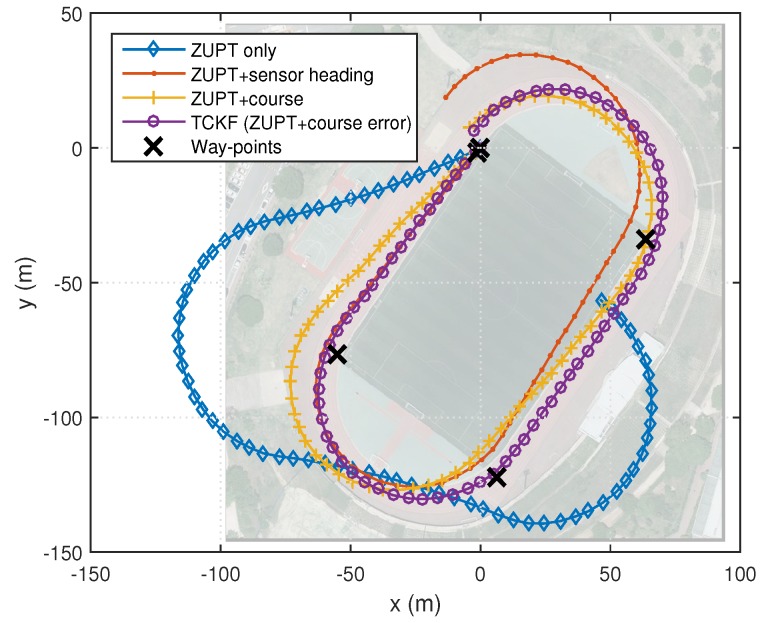
PDR results for the navigation systems on the left foot.

**Figure 8:**
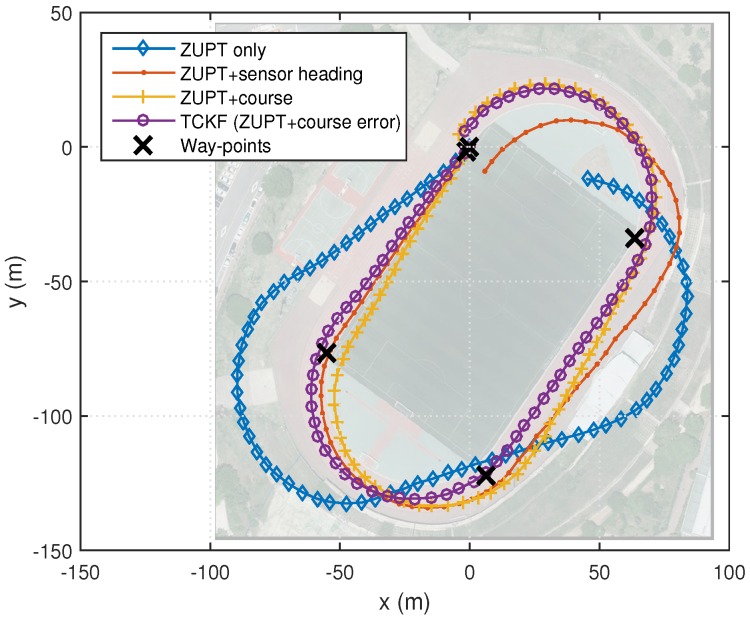
PDR results for the navigation systems on the right foot.

**Figure 9:**
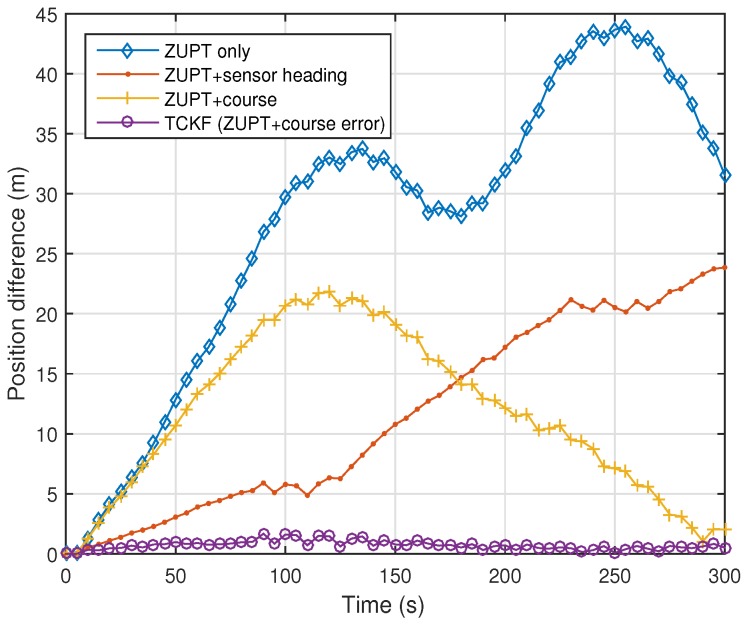
Root-mean-square (RMS) values of position differences between left foot and right foot.

**Figure 10:**
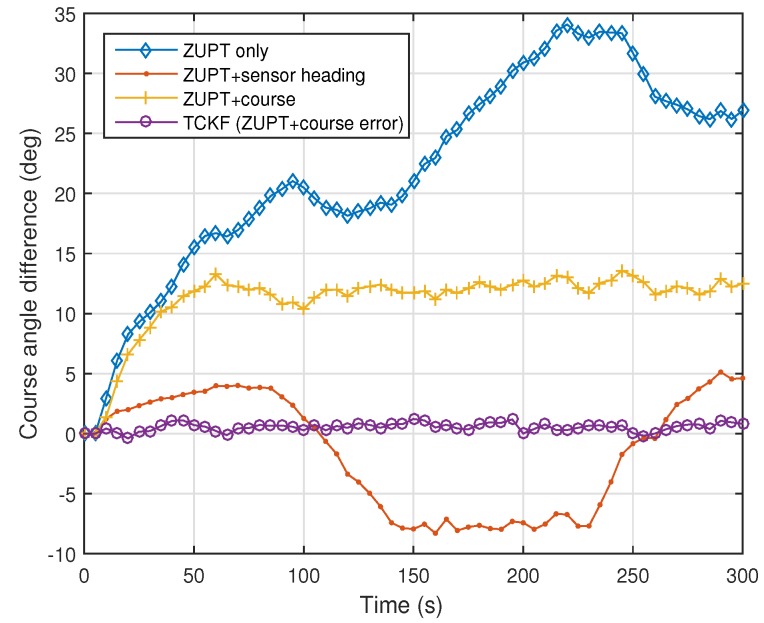
Root-mean-square (RMS) values of angle differences between course angles calculated from the left foot position and those from the right foot position.

The experimental results are shown and summarized in the following figures and table. The walking trajectory is a closed path along a 400-m track, as shown in the first two figures. Figure 7 and Figure 8 show the positions of the left and right feet for four cases, respectively. Figure 9 presents the RMS values of the position differences between the left and right feet, which show the relative position error along the track. The position differences between them are mainly related to the heading or course error of each system. The calculated course angle errors for the left and right feet systems are shown in Figure 10.

These results show that the ZUPT-only cases include a large position error because no heading information is used. In the ZUPT + sensor heading cases, the position of the left foot shows a quite different trajectory from that of the right foot, which is mainly due to the magnetic disturbances imposed on the foot-mounted magnetic sensors and different toe angles. The position trajectories of the ZUPT + course cases are similar to the true trajectory, but they show conspicuous errors along the track. The different toe angles of the left and right feet, which are not considered in these cases, induce the error. Thus, the exact toe angle should be determined or estimated in these cases for acquiring a relatively accurate position solution. However, the toe angle information cannot be easily obtained because it depends on the walking pattern of each individual, walking velocity, sensor alignment errors, road condition, and so on, which results in the performance limitation of the ZUPT + course cases.

**Figure 11:**
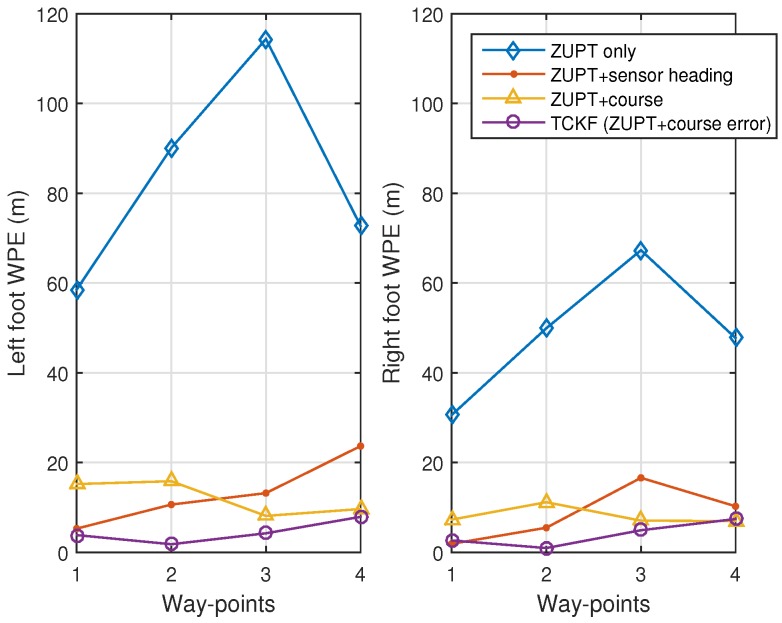
Way-point position errors (WPEs) for left foot and right foot.

The proposed TCKF method, however, provides a relatively accurate position solution, as shown in the figures. In addition to the position accuracy, the position differences between the left foot and right foot are also maintained within 1 m, which is a reasonable result, and the course angle differences are also bounded under $1^{\circ }$ as well. The overall performance enhancement by the proposed algorithm is shown in Figure 11. This figure shows the WPE for each case. From the results, it is confirmed that the proposed algorithm suppresses the position error at every way-point, which implies that the performance is improved by applying the proposed algorithm.

The experimental results are summarized in Table 2. The summarized results also show that the overall performances, including position and course accuracy, are improved dramatically by applying the proposed TCKF algorithm. Particularly, the difference between the left foot position and right foot position is very small compared with other results, which implies that the proposed algorithm can provide a robust solution against installation errors and walking gait differences.

### 4.2. Indoor Cases

**Figure 12:**
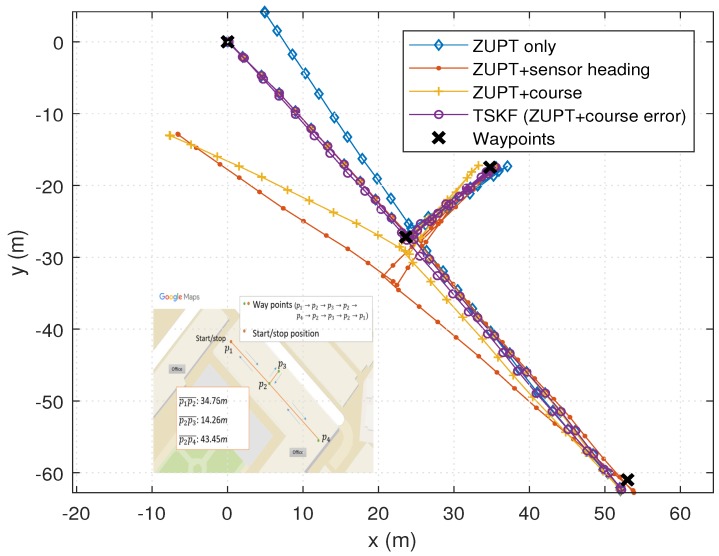
One of the indoor PDR results.

The experimental results for the indoor cases are shown in Figure 12 and Table 3. The experiments were carried out for seven people in several cases, including office buildings, halls, and corridors. The experimental results show the benefit of the proposed algorithm more clearly.

Figure 12 shows the results of one sample case conducted in an office building. In this case, the initial heading was set in advance. The results show that the error is small when applying the proposed algorithm but large when using the existing algorithms. In the ZUPT-only case, the error increases over time because the heading is not corrected at all. Although the error in the experiment result does not seem to be large, it is expected that the error will increase unlimitedly over time, which is dependent on the bias and scale factor error of the gyroscopes.

The ZUPT + sensor heading results show that the trajectory is distorted owing to the magnetic disturbances. The ZUPT + course case is also affected by the magnetic disturbances, but the aspect is slightly different. While the path of the ZUPT + sensor heading method is irregularly bent in the rotation section owing to the excessive magnetic disturbances, the result of the ZUPT + course method exhibits a coherent path error that bends to the right slightly, which is due to the toe angle and magnetic disturbances. That is, the error patterns of the ZUPT + course cases are also related to the human walking habits, walking speed, and gait patterns, along with the magnetic disturbances. Nevertheless, both results show the same error characteristics, which are not significantly related to time because the heading error is compensated, even though it is incomplete. Thus, the error appears repeatedly in the same pattern when the experimenter repeats the same path. The proposed TCKF method, on the other hand, shows little WPE despite the magnetic disturbances.

The indoor experiment results summarized in Table 3 show that the proposed algorithm guarantees the smallest WPE errors compared with other methods. They also show that the variance of the errors is the smallest when applying the proposed TCKF algorithm, which means that the proposed algorithm is robust to the magnetic disturbances and gait characteristics of the pedestrians. The errors in the ZUPT-only case have larger deviations because they depend on the gyroscope bias and scale factor errors. The errors in the case of the ZUPT + sensor heading are the largest, but their deviation is not relatively large because they are more influenced by the excessive magnetic disturbances around the building floor than by the errors of the gyroscopes. The results obtained by applying the ZUPT + course method have the largest deviation of errors because they are sensitive not only to the magnetic disturbances but also to the gait characteristics of each person. From the results obtained by the indoor experiments, it is confirmed that the proposed algorithm is more effective and robust against the magnetic disturbances and various gait characteristics.

In this research, the height errors are not taken into account because we focus more on the heading angle accuracy and two-dimensional position accuracy determined mainly by it. However, the experiments involving magnetic disturbances and heading accuracy include stair cases, so we can make similar conclusions from experiments on the three-dimensional paths as far as the heading accuracy and two-dimensional position accuracy are concerned.

## 5. Conclusions

In this study, a pedestrian navigation algorithm based on a TCKF was proposed. The first filter in the TCKF estimates the course error between the magnetic heading of the waist-mounted sensors and the walking course, and the second filter estimates other navigation errors using the ZUPT.

In the first filter, the walking course predicted by using the position trace is assumed to be similar to the direction of the body, and the magnetic heading of the waist-mounted sensors is used for generating measurements. Using these measurements, the course error is estimated first. In the second stage filter, the estimated course error is used for estimating the heading error of the foot-mounted sensors. It is noted that the heading error of the foot-mounted sensors induces the walking course error. Thus, we can assume that the course error is similar to, and even almost the same as, the heading error of the foot-mounted sensors, though the course of a body is quite different from the heading of the foot-mounted sensors. Therefore, the proposed TCKF can estimate and correct the heading error indirectly by applying the concept of course error.

The effectiveness and performance improvement achieved by the proposed method are confirmed through indoor and outdoor experiments. The experimental results show that the position accuracy is improved by maximum 90% compared with the conventional ZUPT-based pedestrian navigation algorithm. Moreover, the robustness of the proposed algorithm against the installation error and walking gait characteristics is also validated through a comparison between the left foot and right foot positions. The performance improvements are more effectively shown by the experiments performed in various indoor cases by several people. In order to further improve the performance, the misalignment error of the waist-mounted magnetic sensors should also be considered or pre-determined, which will be a topic of further research. Moreover, other sensor fusion algorithms for height estimation should be taken into account for improving the three-dimensional position accuracy.

## Figures and Tables

**Table 1:** Indoor magnetic heading errors.

Sites	Number of Sites	RMS Error around Waist	RMS Error around Foot	Error Ratio
Office buildings	15	4.993	12.995	2.60
Home buildings	10	8.442	17.579	2.08
Halls/malls	15	6.854	19.408	2.83
Underground spaces	10	7.778	20.937	2.69
Mean		7.017	17.734	2.53

**Table 2:** Summary of experimental results.

	ZUPT-only	ZUPT + Sensor Heading	ZUPT + Course	TCKF (ZUPT + Course Error Estimation)
RPE of left foot (m)	73.161	22.995	8.671	6.595
RPE of right foot (m)	46.836	10.751	6.318	5.994
Position difference (RMS, m)	30.491	13.963	13.437	0.794
Course difference (RMS, ${}^{\circ }$)	23.592	5.082	11.542	0.626
WPE of left foot (mean, m)	83.933	13.208	12.218	4.461
WPE of right foot (mean, m)	48.894	8.568	8.130	3.999

**Table 3:** Summary of experimental results (indoor case).

Average WPE (m)	ZUPT Only	ZUPT + Sensor Heading	ZUPT + Course	TCKF (ZUPT + Course Error Estimation)
Case 1	2.561	3.776	3.201	1.237
Case 2	2.917	3.918	2.594	0.969
Case 3	5.091	4.403	7.107	1.396
Case 4	1.407	4.071	1.085	0.822
Case 5	0.989	5.569	6.280	0.864
Case 6	2.666	3.057	1.954	0.691
Case 7	2.373	5.482	5.365	0.871
Mean	2.572	4.325	3.941	0.978
Standard deviation	1.315	0.916	2.309	0.250
